# Transcriptional trajectories of anther development provide candidates for engineering male fertility in sorghum

**DOI:** 10.1038/s41598-020-57717-0

**Published:** 2020-01-21

**Authors:** Namrata Dhaka, Kushagra Krishnan, Manu Kandpal, Ira Vashisht, Madan Pal, Manoj Kumar Sharma, Rita Sharma

**Affiliations:** 1 Crop Genetics & Informatics Group, School of Computational and Integrative Sciences, Jawaharlal Nehru University, New Mehrauli Road, New Delhi, 110067 India; 2 Division of Plant Physiology, Indian Agricultural Research Institute, Pusa, New Delhi, 110012 India; 3 Crop Genetics & Informatics Group, School of Biotechnology, Jawaharlal Nehru University, New Mehrauli Road, New Delhi, 110067 India

**Keywords:** Transcriptomics, Pollen

## Abstract

Sorghum is a self-pollinated crop with multiple economic uses as cereal, forage, and biofuel feedstock. Hybrid breeding is a cornerstone for sorghum improvement strategies that currently relies on cytoplasmic male sterile lines. To engineer genic male sterility, it is imperative to examine the genetic components regulating anther/pollen development in sorghum. To this end, we have performed transcriptomic analysis from three temporal stages of developing anthers that correspond to meiotic, microspore and mature pollen stages. A total of 5286 genes were differentially regulated among the three anther stages with 890 of them exhibiting anther-preferential expression. Differentially expressed genes could be clubbed into seven distinct developmental trajectories using K-means clustering. Pathway mapping revealed that genes involved in cell cycle, DNA repair, regulation of transcription, brassinosteroid and auxin biosynthesis/signalling exhibit peak expression in meiotic anthers, while those regulating abiotic stress, carbohydrate metabolism, and transport were enriched in microspore stage. Conversely, genes associated with protein degradation, post-translational modifications, cell wall biosynthesis/modifications, abscisic acid, ethylene, cytokinin and jasmonic acid biosynthesis/signalling were highly expressed in mature pollen stage. High concurrence in transcriptional dynamics and *cis*-regulatory elements of differentially expressed genes in rice and sorghum confirmed conserved developmental pathways regulating anther development across species. Comprehensive literature survey in conjunction with orthology analysis and anther-preferential accumulation enabled shortlisting of 21 prospective candidates for in-depth characterization and engineering male fertility in sorghum.

## Introduction

Sorghum, grown widely for food, feed, and forage, is a gluten-free substitute for staple grains, and a promising feedstock for biofuels^1^. It exhibits huge phenotypic and morphological diversity in key agronomic traits including photoperiod sensitivity, biomass, grain yield, disease resistance, abiotic stress tolerance etc., with considerable scope for genetic enhancement of cultivated sorghum. However, sorghum is a self-pollinated crop with 75 to 95% rate of self-pollination observed under natural conditions with outcrossing rates varying with panicle type, distance between plants, wind direction, etc^2,3^. Therefore, overcoming reproductive constraints is a major challenge to utilize sorghum diversity for breeding programs.

Plant breeders have been using cytoplasmic (CMS) and nuclear male sterility (NMS) systems to take advantage of hybrid vigour in many self-pollinated crop species^4^. While CMS, caused by specific nuclear and mitochondrial genetic interactions, is maternally inherited; NMS results due to defects in nuclear genes usually inherited as a recessive trait^5^. Studies in rice suggest that hybrids produced through NMS give higher yields and germplasm utilization efficiency with better genetic stability under diverse environmental conditions as compared to those produced through CMS^6^. However, hybrid production in sorghum exclusively relies on the CMS system where male sterile line is crossed with an identical male fertile line leading to hybrids with sterile seeds. These are then crossed with a restorer line to restore fertility^5^. This entire process is very time consuming and laborious. Also, repeated use of cytoplasm from male-sterile line is detrimental for plant fitness and adaptability in the long run^6^. Although NMS is an easier alternative, there is paucity of information about NMS-related genes in sorghum^7^. So far, only one NMS mutant *ms8* has been characterized in sorghum that can be visually identified due to shorter anthers lacking pollen^8^. Leveraging NMS system in sorghum needs active exploration of anther transcriptome, followed by identification of key genes regulating male fertility.

Christensen^9^ used light and electron microscopy to examine developmental progression of sporogenous tissue to mature pollen in sorghum anthers. However, transcriptional dynamics in sorghum anthers remain unexplored. In this study, we identified key landmark events and generated a transcriptional landscape from three successive stages of sorghum anther development using RNA sequencing. A total of 1.08 billion high quality reads were generated from nine libraries with an average read length of 100 bp. Out of 31871 genes expressing in sorghum anthers, 5286 genes differentially expressed in different stages of anther development, were categorized into seven distinct transcriptional trajectories based on their expression patterns. Pathway analysis and mapping of orthologs, from model species rice and *Arabidopsis*, highlighted conserved as well as unique genetic components underlying progression of male gametophyte development in sorghum. The shortlisted candidates provide important targets for engineering male sterility in sorghum.

## Results and Discussion

### Anther staging and global dynamics of gene activity

**Figure 1:**
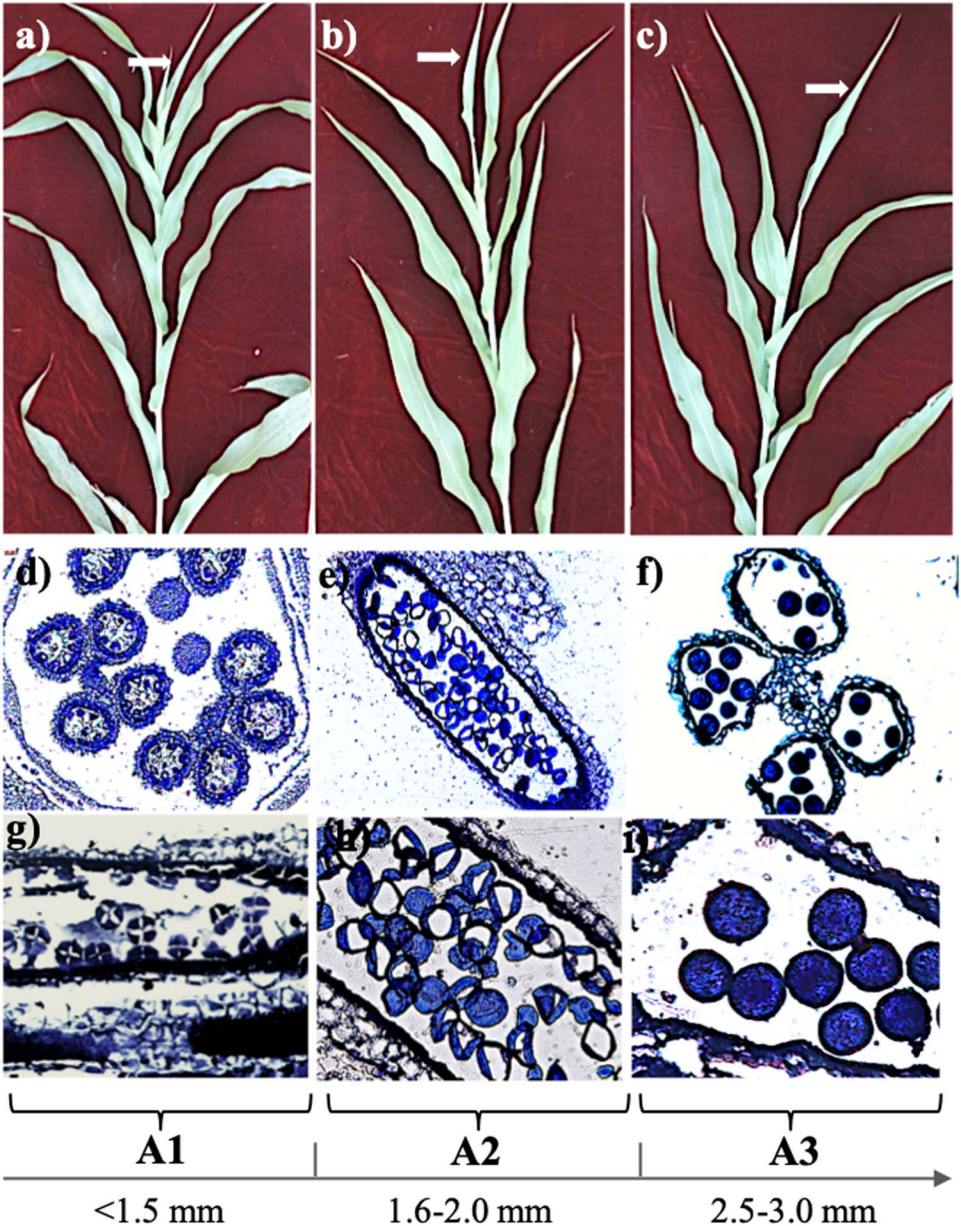
Staging of sorghum anthers. The position of flag leaf, shown by a white arrow, varying from partial to complete emergence in (**a**) A1, (**b**) A2, and (**c**) A3 stages was used as indicator of stage of development. Sections of florets representing A1 (**d**,**g**), A2 (**e**,**h**) and A3 (**f**,**i**) stages at 100× and 400× magnification, respectively.

Length of anthers is tightly correlated with the key developmental events in sorghum^9^. Leveraging this information, we collected sorghum anthers at three temporal stages corresponding to three distinct landmark stages viz., meiotic to tetrad (A1; <1.5 mm), tetrad to microspore (A2; 1.6–2.0 mm), and mature pollen (A3; 2.5–3.0 mm). The position of flag leaf was used as a visual indicator to identify the stage of panicle development (Fig. 1). Concurrently, anthers were sampled from panicles collected from various stages of pre-emergence and post emergence panicle for microscopic analysis. In agreement with the earlier report^9^, microscopic analysis confirmed meiotic stages and tetrads in stage A1. Stage A2 contained microspores, whereas, anthers at stage A3 harboured mature engorged pollen (Fig. 1).

**Figure 2:**
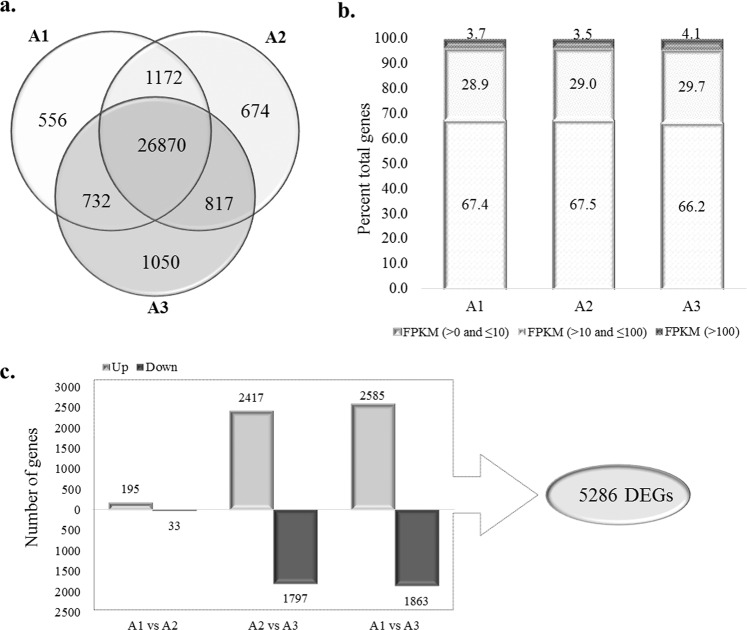
Expression dynamics of sorghum anthers. (**a**) Venn diagram showing overlap between expressed genes in all three stages; (**b**) Distribution of FPKM range in all three stages of anther development and (**c**) Results of differential expression analysis.

Sequencing of nine libraries (three replicates from each stage) yielded >1.12 billion paired-end reads. After filtering at Q30 and adapter trimming, 1.08 billion high-quality reads were obtained. These reads were further filtered to remove rRNA and finally >961 million high-quality reads could be aligned to the sorghum genome (Phytozome V12 Sbicolor 313 v3.0.1; Table S1). A total of 32891 unique genes were determined by using STAR alignment. Fragments per kilobase per million reads (FPKM) were determined for all the unique transcripts. A total of 31871 genes with FPKM >0, in all three replicates of at least one anther stage, were considered as expressed. The expression of these genes was highly correlated among the biological replicates of each stage, with an average Spearman’s correlation coefficient of 0.91 among biological replicates (Table S2). Among the 31871 genes, 26870 genes expressed in all three stages, while 556, 674, and 1050 genes each were unique to A1, A2, and A3 stages, respectively (Fig. 2a). Comparative analysis revealed maximum overlap between A1 and A2 stages with 1172 genes expressing in these two stages, 817 genes common in A2 and A3 stages, while 732 genes were common in A1 and A3 stages (Fig. 2a). Analysis of transcript abundance in all three stages revealed 66.2 to 67.5% genes exhibiting low expression (FPKM >0 to ≤10), 28.9 to 29.7% genes with moderate expression (FPKM >10 to ≤100), whereas, merely 3.5 to 4.1% genes showed high expression with FPKM >100 (Fig. 2b). Evidently, A3 stage containing mature pollen contains largest number of unique transcripts and genes with high abundance.

Pairwise differential expression analysis between all three stages resulted in a set of 5286 differentially expressed genes (DEGs) with ≥2-fold change at q value ≤0.05 (Fig. 2c). Among all comparisons, highest number of DEGs were in the A3 stage with 4448 genes (2585 up and 1863 downregulated) differentially expressed compared to A1 stage and, 4214 (2417 up and 1797 down regulated) genes differentially expressed in comparison to A2 stage. The A1 and A2 stages shared transcriptional repertoire with only 228 genes differentially expressed (195 up and 33 down regulated) in A2 with respect to A1 (Fig. 2c). These results further confirm that mature pollen stage has more complex and distinct expression profile compared to early developmental stages. Further, we used the publicly available data from 47 sorghum RNA-sequencing experiments in Phytozome to identify anther-preferential genes. After filtering genes with FPKM ≥1 in any of the vegetative or seed tissues, 890 of the differentially expressed genes were found to exhibit anther-preferential accumulation. These play role in regulating cell division, cell wall, development, DNA repair & synthesis, transcription, transport, stress, signalling and metabolic processes including amino acid, carbohydrate, lipid and protein metabolism (Table S3A).

### Dominant patterns of gene activity and associated genes/pathways

**Figure 3:**
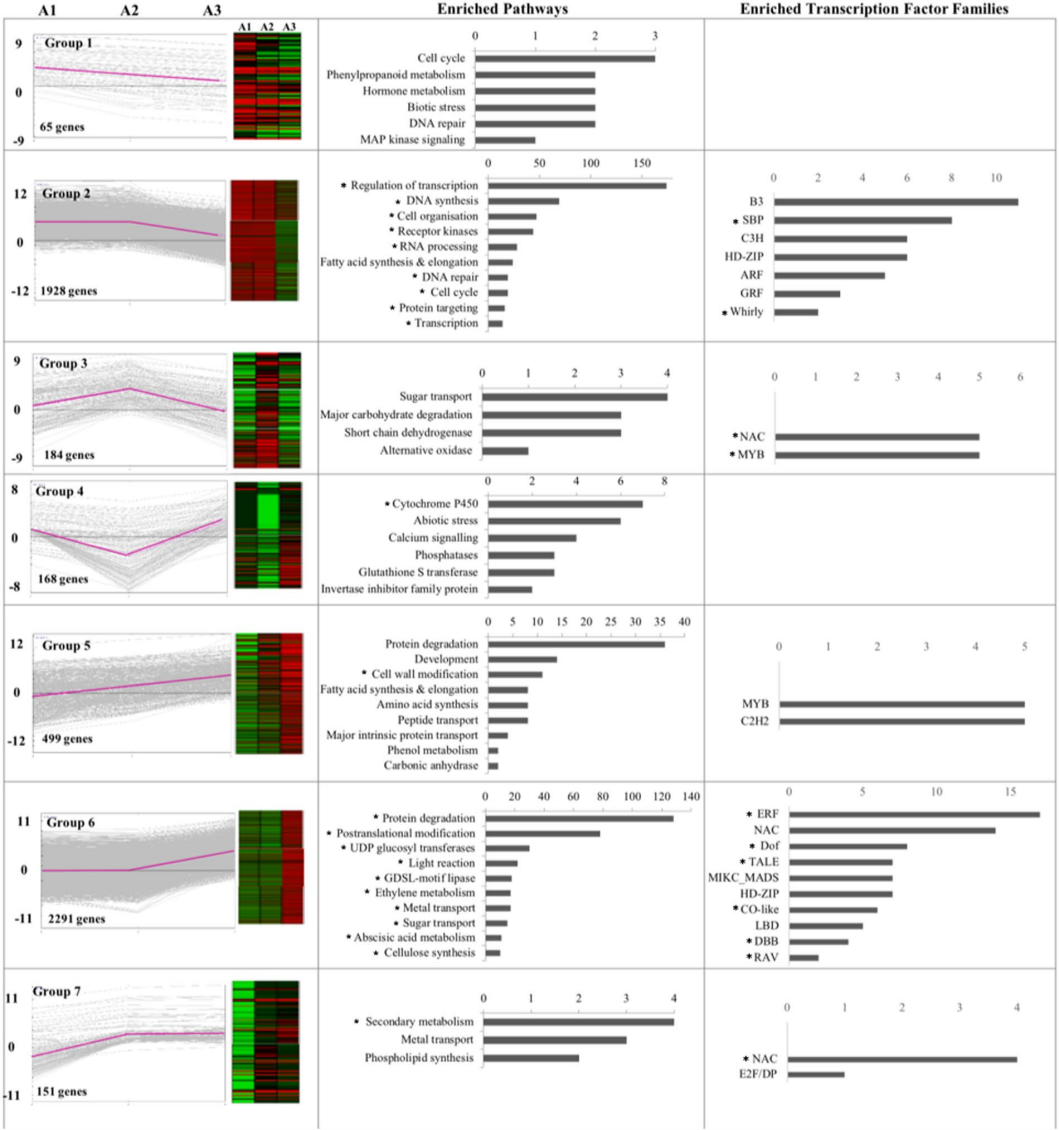
Dominant patterns exhibited by differentially expressed genes (DEGs), and enriched pathway sub-categories and transcription factor families. Grouping of DEGs was done based on K-means clustering and identification of enriched pathway sub-categories and transcription factor families was carried out using hypergeometric test (p-value ≤ 0.05). Top 10 pathway sub-categories have been shown for the groups containing more than 10 pathways. The asterisks are used to mark the sub-categories for which the p-value for enrichment was significant after multiple correction with Benjamini-Hochberg test (FDR < 0.05).

Identification of dominant expression patterns associated with developmental progression is a key step in deciphering co-regulated gene clusters. A total of sixteen clusters were obtained from 5286 DEGs using K-means clustering. Subsequently, gene clusters that only differed in the magnitude of expression were combined together resulting in seven distinct transcriptional trajectories, referred hereafter, as ‘groups 1–7’ (Fig. 3, Table S3). Only four genes (Sobic.001G189300, Sobic.004G189500, Sobic.006G034300 and Sobic.004G033500) showed decrease in expression from A1 to A2 stage with no significant difference in A3 stage. Likely due to small number, these were clubbed with group 1 during clustering.

It is noteworthy that ~80% of the genes belonged to groups 2 and 6, with uniform expression levels in meiotic (A1) to post-meiotic (A2) stages followed by either decline (group 2) or increase (group 6) in the mature pollen (A3) stage. To investigate the functional relevance of these patterns, we used MapMan to map the genes belonging to all seven groups onto biological pathways. Out of 5286 DEGs, 4221 genes mapped to 34 MapMan pathways. Also, 337 of the DEGs mapped to transcription factors. Analysis of over-enrichment of all sub-categories of pathways and transcription factor families was performed using hypergeometric test with a p value cut-off of ≤0.05 and FDR <0.05 (Tables S4 and S5). The detailed functional assessment of each group is elaborated below:

#### Group 1 genes putatively regulate male meiosis

Group 1 contains 65 genes with peak expression in the meiotic (A1) stage that gradual declines in post-meiotic microspore (A2) and mature pollen (A3) stages (Fig. 3). Pathway mapping revealed several genes mapping to cell cycle, biotic stress, DNA repair, hormone metabolism, phenylpropanoid metabolism, and MAP kinase signalling pathways in this group (Fig. 3, Table S4). Five of the genes in this group exhibit anther-preferential expression (Sobic.002G158300, Sobic.002G263000, Sobic.003G211300, Sobic.003G347100, Sobic.004G204400), however, none of these genes or their orthologs have been experimentally shown to play role in anther development. Hormone-related genes in this group including 1-aminocyclopropane-1-carboxylate oxidase involved in ethylene biosynthesis, and gibberellin 2-oxidase which inactivates endogenous gibberellins highlight the role of ethylene and gibberellins in meiotic progression^10^. Ethylene production has earlier been associated with degeneration of tapetal cells and middle layers of anther wall in petunia^11^. Empirical studies will be required to examine if ethylene plays similar role in sorghum anthers as well. Some of the genes relevant to cell cycle such as Sobic.001G462300 and Sobic.008G104000 (orthologous to *Arabidopsis Cyclin A3* and *Cyclin A2*, respectively) also exhibit group 1 pattern. These may also be associated with male meiosis.

Rice orthologs of two genes from this group, Sobic.008G010900 (orthologous to *OsDMC1*; *disrupted meiotic cDNA1*) and Sobic.002G003300 (orthologous to *Osku70; ATP-dependent DNA helicase 2 subunit KU70*) have already been shown to play role in male meiosis (Table 1). *OsDMC1* is essential for homologous pairing, whereas, *Osku70* maintains chromosomal stability during meiosis in pollen mother cells^12,13^.

**Table 1:** List of shortlisted candidates for in-depth characterization.

S. No.	Sorghum gene	Rice/Arabidopsis Ortholog	Function
**Group 1: Peak expression at A1**			
1	Sobic.008G010900	*DMC1*	Homologous pairing during meiosis^12^
2	Sobic.002G003300	*OsKu70*	Chromosome stability during meiosis^13^
**Group 2: Peak expression at A1 and A2**			
3	^*^Sobic.002G304500	*OsMSH5*	Meiotic crossover^107^
4	Sobic.006G218400	*Ctb1*	Anther length^108^
5	Sobic.006G185600	*HTH1*	Anther wall development^40^
6	^*^Sobic.004G101500	*HEI10*	Chiasmata formation^19^
7	Sobic.010G191000	*OsCOM1*	Chromosome pairing & synapsis^109^
8	Sobic.006G120600	*OsCRC1*	Chromosome pairing & synapsis^110^
9	Sobic.002G074500	*OsSGO1*	Chromosome pairing & synapsis^111^
10	Sobic.004G063300	*PAIR3*	Chromosome pairing & synapsis^112^
11	Sobic.002G353500	*OIP30*	Detected in prophase I^24^
12	^*^Sobic.001G491400	*CYP704B2*	Exine & anther cuticle development^113^
13	^*^Sobic.007G180700	*LTPL44*	Exine development^35^
14	Sobic.001G207600	*OsABCG26*	Exine development^6^
15	Sobic.007G029900	*CYP703A3*	Exine development & anther cuticle development^32^
16	Sobic.002G115700	*OsPKS2*	Exine development & ubisch body patterning^114^
17	Sobic.002G368600	*UAM3*	Intine development^38^
18	Sobic.001G507000	*MEICA1*	Meiotic crossover^115^
19	Sobic.006G231800	*MRE11*	Meiotic crossover^25^
20	^*^Sobic.009G185700	*OsMSH4*	Meiotic crossover^27^
21	Sobic.009G012600	*RPA1c*	Meiotic crossover^26^
22	Sobic.007G149550	*RPA1c*	Meiotic crossover^26^
23	Sobic.004G355500	*RPA2A*	Meiotic crossover^26^
24	Sobic.010G243100	*RPA2C*	Meiotic crossover^26^
25	Sobic.006G101800	*ZEP1*	Meiotic crossover^29^
26	Sobic.001G415800	*ZEP1*	Meiotic crossover^29^
27	Sobic.007G097000	*OsRAD21*	Regulates meiosis & microspore mitosis^116^
28	^*^Sobic.007G164101	*MEL2*	Regulation of meiotic genes^117^
29	^*^Sobic.002G270400	*LTPL45/YY1*	Specifically expressed in uninucleate microspore^35^
30	^*^Sobic.003G140100	*TIP2*	Tapetum degeneration, pollen development & anther dehiscence^20^
31	^*^Sobic.001G428300	*OsSTRL2*	Tapetum degeneration & pollen development^34^
32	^*^Sobic.006G079500	*OsACOS12*	Tapetum degeneration, anther cuticle & pollen wall development^118^
33	Sobic.008G008100	*DTC1*	Tapetum degneration^39^
34	Sobic.001G478100	*OsAP25*	Tapetum degneration^21^
35	Sobic.003G328500	*OsCycB1*	G2/M phase specific gene^28^
36	Sobic.002G056000	*MET1*	Meiotic regulation^18^
37	Sobic.004G047500	*AtWHIRLY2*	Binding of mtDNA in developing pollen^41^
38	Sobic.010G215700	*SPL8*	Anther wall development^119^
39	Sobic.010G254200	*SPL2*	Anther wall development^42^
40	Sobic.004G051900	*ARF8*	Anther dehiscence^120^
**Group3: Peak expression at A2**			
41	^*^Sobic.001G099700	*OsINV4*	Confers cold tolerance to pollen^49^
42	Sobic.004G181500	*XRCC3*	Double strand break repair & homologous recombination^46^
43	^*^Sobic.004G017500	*TDR*	Tapetum degeneration^20^
44	^*^Sobic.001G320300	*OsNP1*	Regulated tapetum degradation & exine formation^6^
45	^*^Sobic.001G266600	*OsDIL*	Pollen fertility in drought^48^
**Group 4: Peak expression at A1 and A3**			
46	Sobic.007G156300	*OsABA8Ox3*	Regulates cold induced pollen sterility^50^
47	Sobic.003G365600	*MID1*	Regulates anther development in drought stress^53^
**Group 5: Peak expression at A3**			
48	^*^Sobic.004G100900	*OsPCBP*	Calcium signalling during starch accumulation in pollen^63^
49	^*^Sobic.001G306400	*OSIPA*	Pollen expansin^62^
50	^*^Sobic.001G306200	*OSIPA*	Pollen expansin^62^
51	Sobic.006G255600	*OsINV1*	Confers cold tolerance to pollen by regulating sucrose accumulation^49^
52	Sobic.009G154200	*MID1*	Regulates anther development in drought stress^53^
**Group 6: Peak expression at A3**			
53	^*^Sobic.008G138200	*MEL2*	Regulation of meiotic genes^117^
54	Sobic.004G149100	*DAO*	Regulates anther dehiscence & pollen fertility by auxin homeostasis^121^
55	Sobic.009G012500	*RPA1c*	Meiotic crossover^26^
56	Sobic.009G012200	*RPA1c*	Meiotic crossover^26^
57	^*^Sobic.003G027000	*OsMADS3*	Regulates ROS homeostasis & impacts pollen fertility^122^
58	^*^Sobic.006G159000	*OsPLIM2b*	Anther specific gene interacts with male sterility related protein kinase OsNek3^76^
59	Sobic.004G013500	*OsUgp2*	Pollen starch accumulation^68^
60	Sobic.002G125900	*ORMDL*	Regulates sphingolipid homeostasis^123^
61	Sobic.001G222700	*WDA1*	Fatty acid synthesis in exine & anther cuticle^69^
62	^*^Sobic.006G070032	*OsINV4*	Confers cold tolerance to pollen^49^
63	Sobic.006G118400	*DAO*	Regulates anther dehiscence & pollen fertility by auxin homeostasis^121^

*Genes marked with asterisk exhibit anther-preferential expression.

#### Group 2 genes regulate male meiosis, tapetum degradation and pollen wall formation

Group 2 comprises 1928 genes with high expression in both meiotic and post-meiotic stages and prominent decline in mature pollen stage (Fig. 3). A total of 12 pathway sub-categories were represented in this group with ‘regulation of transcription’ as the most abundant, followed by DNA synthesis/chromatin structure, cell organization, receptor kinases, RNA processing, DNA repair, cell cycle, protein targeting, RNA binding, metabolite transporters, and amino acid activation (Fig. 3, Table S4). Apart from the above categories, several genes associated with brassinosteroid as well as auxin biosynthesis and signalling fall in this group (Table S3b). Brassinosteroid biosynthesis and signalling has earlier been associated with tapetum and microspore development in *Arabidopsis*^14^.

Also, several genes encoding transcriptional regulators like Argonautes (AGOs), chromatin remodelling factors, DNA methyltransferases, histone acetyl/deacetyl transferases, chromatin assembly factors, aspartic proteases, etc. were also enriched in this group pointing to the role of RNA silencing machinery and epigenetic modifications in male gametophyte development. Sobic.009G259900 and Sobic.003G129500 are orthologs of rice *OsAGO4b* and *OsAGO4a*, respectively, involved in gene silencing through miRNA-mediated cleavage or DNA methylation^15^. Also, Sobic.001G153600 is orthologous to *OsAGO18*, which regulates production of phasiRNAs in grass anthers^16^. While the exact mechanism of action of phasiRNAs has not been deciphered so far, their importance in the male gametophyte development has been emphasised in both rice and maize^17^. Another important candidate gene Sobic.002G056000, orthologous to *Arabidopsis MET1* (Methyltransferase) regulates meiosis during male gametogenesis^18^. Furthermore, rice orthologs of three genes in this group have been shown to perform important roles in chiasmata formation (Sobic.004G101500) (*HEI10*; *Human enhancer of invasion-*10^19^) and tapetum degeneration (Sobic.003G140100 orthologous to *TDR interacting protein 2*; *TIP2*^20^ and Sobic.001G478100 orthologous to *Aspartic Protease 25; OsAP25*^21^) (Table 1). Several genes encoding proteins involved in DNA synthesis/chromatin structure including exonucleases, ATP binding proteins, RNA helicase, cell cycle checkpoint proteins, chromosome condensation, and segregation proteins, DNA gyrase, topoisomerases, helicases, etc. are also enriched in group 2.

Overall, rice orthologs of 37 genes of this group have been previously demonstrated to regulate male gametophyte development using transgenic approaches (Table 1). These include sorghum ortholog (Sobic.007G097000) of *OsRAD21-3* (*Radiation sensitive mutant 21-3*) that regulates pollen mitosis^22^. Transcripts of rice ortholog (*LTPL44; Lipid Transfer Family Protein 44*) of Sobic.007G180700 are also abundant in tetrads and uninucleate microspores with anticipated role in lipid transport in developing pollen^23^. Sobic.002G353500 is orthologous to rice *OIP30* (*OsCPK25/26-interacting protein 30*), which codes for a putative ruvB-like 2 protein, detected during early prophase I in meiosis^24^. DNA repair genes, Sobic.010G243100, Sobic.004G355500, Sobic.006G231800, and Sobic.009G185700, are orthologous to rice DNA repair genes important for meiotic crossovers namely, *RPA2c* (*replication protein 2c*), *RPA2a* (*Replication Protein 2a*), *MRE11*(*meiotic recombination 11*), and *OsMSH4* (*Mut S Homolog 4*), respectively^25–27^.

Cell cycle-related genes encoding cyclins, tetratricopeptide repeat containing proteins, auxin response factors, cyclophilins, and peptidyl-prolyl isomerases are also prominent in this group. Rice ortholog (*OsCycB1*) of one of the cyclin genes, Sobic.003G328500, is known to exhibit G2/M phase-specific expression in rice^28^. Sobic.006G101800 is ortholog of *ZEP1*, a putative synaptonemal complex protein, which regulates the number of crossovers during meiosis^29^.

Several lipid metabolism-related genes encoding acetyl-CoA carboxylase, acetyl-CoA transacetylase, ACP desaturases, ACP proteins, acyl CoA ligases, acyl-CoA binding protein, beta hydroxyacyl ACP dehydratase, beta ketoacyl-CoA synthases, enoyl ACP reductase, ketoacyl ACP synthases (KCS), and long chain fatty acid CoA ligases are also prominent in this group. These are important candidates for regulating pollen wall formation. In fact, in *Arabidopsis*, several KCS genes have been already implicated in sporopollenin synthesis and pollen wall formation^30^.

Receptor kinases or receptor-like kinases (RLKs) also play prominent role in sensing extracellular signals and have also been implicated in pollen development^31^. Out of 44 receptor kinases present in group 2, 25 are leucine-rich repeat XI types while 8 are leucine-rich repeat III types. Another important gene in this subset is Sobic.007G029900 which is orthologous to rice *CYP703A3* (*Cytochrome P450 Hydroxylase 703A3*), recently shown to be the causal gene for the *no-pollen* male sterility mutant in *indica* rice^32,33^.

Rice orthologs of several other genes in this group are involved in regulating key aspects of anther development including chromosome pairing and synapsis (*Completion of meiosis1; OsCOM1*, *Central region component 1; OsCRC1*, *OsSGO1* and *Homologous Pairing Aberration in Rice Meiosis 3; PAIR3*), meiotic crossover (*Meiotic chromosome association1; MEICA1*, *RPA1c*, *ZEP1* and *OsMSH5*), pollen wall development (*Cytochrome P450 Hydroxylase 704B2; CYP704B2*, *LTPL44*, *CYP703A3*, *ATP Binding Cassette G26; OsABCG26*, *Polyketide synthase2; OsPKS2* and *UDP-arabipyranose mutase3; UAM3*), anther wall development (*HTH1* (*Hothead1*)), meiotic regulation (*MEL2*), and tapetum degeneration (*STRL-like2; OsSTRL2*, *Acyl CoA synthatase12; OsACOS12*, and *Defective Tapetum Cell Death1; DTC1*) (Table 1).

Out of 1928 group 2 genes, 231 exhibit anther-preferential expression. Rice orthologs of 10 anther-preferential genes (*OsSTRL2*, *CYP704B2*, *LTPL45*, *OsMSH5*, *TIP2*, *HEI10*, *OsACOS12*, *MEL2*, *LTPL44*, and *OsMSH4*) have also been shown to play important roles in male gametophyte development (Table 1). Sobic.001G428300 is orthologous to *OsSTRL2* that regulates pollen wall formation^34^. Sobic.002G270400 is ortholog of rice *LTPL45* that specifically expresses in the uninucleate microspore stage of rice^35^. Zhao and co-workers^36^ reported identification of *OsABCG26* as the gene responsible for a male sterile mutant in *japonica* rice. It helps in the transport of lipids from tapetal cells for anther cuticle development. Another anther-specific gene *OsPKS2* has recently been shown to be causal gene for a rice male sterile mutant^37^. This gene is crucial for pollen development and sporopollenin biosynthesis in both rice and *Arabidopsis* and therefore, its ortholog Sobic.002G115700 is also a suitable candidate for engineering male sterility in sorghum. Similarly, Sobic.006G079500 is ortholog of *ACOS5* (*Acetyl-CoA synthetase 5*) required for sporopollenin biosynthesis. Another candidate Sobic.002G368600 is orthologous to rice *UDP-arabinopyranose mutase 3* (*UAM3*), which is required for pollen intine development^38^. Furthermore, rice ortholog of Sobic.008G008100, *Defective Tapetum Cell Death1* (*DTC1*), is a key regulator of tapetum programmed cell death^39^, while, Sobic.006G185600 corresponds to rice *HOTHEAD-Like HTH1* which regulates biosynthesis of cutin monomers in the anther epidermis. It has recently been shown that apart from tapetum, epidermal cutin synthesis is also crucial for pollen fertility^40^.

Transcription factors (TFs) in this group mainly belong to ARF (Auxin Response Factors), B3, C3H, GRF (Growth Regulating Factor), HD-ZIP (Homeodomain-leucine zipper), SBP (Squamosa Binding Protein), and Whirly families, with the latter two families showing significant enrichment (Fig. 3). Interestingly, sorghum has two putative Whirly TFs with both (Sobic.010G035200 and Sobic.004G047500) showing group 2 expression pattern. One of these, Sobic.004G047500, is orthologous to *Arabidopsis Whirly2*, crucial for pollen development^41^. SBP factors are also significantly enriched in this group. In fact, none of the other groups apart from group 2 contain SBP TFs. SBP TF genes, Sobic.010G215700 and Sobic.010G254200, are orthologous to *Arabidopsis SPL8* (*Squamosa promoter-binding-like 8*) and *SPL2* (*Squamosa promoter-binding-like 2*), respectively. Both these genes are known to regulate anther development in a redundant manner^42^. Besides SBPs, ARFs have also been previously associated with pollen development. Sobic.004G051900 of this group is orthologous to *Arabidopsis ARF8* (*Auxin response factor 8*), involved in anther development^43^. Sorghum ortholog of *ARF17* (*Auxin response factor 17*) that has been shown to regulate primexine deposition also exhibits group 2 expression pattern^44^. Overall, large number of candidates from this group have been shown to regulate male development confirming high relevance of this group in engineering male fertility.

#### Group 3 genes regulate microspore development

Group 3 contains 184 genes with peak expression in A2 stage containing developing microspores (Fig. 3). This group contains many sugar transporters and genes involved in carbohydrate degradation (Fig. 3, Table S4). Analysis of characterized rice orthologs of sorghum genes belonging to group 3 highlighted several important candidates for microspore development (Table 1). For instance, rice ortholog of Sobic.001G320300, *OsNP1* (*No Pollen 1*), regulates tapetum degradation and exine formation; Sobic.004G017500 ortholog, *TDR* (*Tapetum Degeneration Retardation*), is a master regulator of anther development in both rice and *Arabidopsis*^45^, whereas, Sobic.004G181500 ortholog *XRCC3* (*X-ray repair cross-complementing group 3*), regulates double strand break repair and homologous recombination during meiosis in rice^46^. Another interesting candidate from this group Sobic.008G016500 encodes for a putative rhamnogalacturonate lyase. Rice ortholog of this gene (LOC_Os12g03790) has been implicated in dissociation of microspores from tetrads through pectin degradation^47^.

Several rice orthologs of sorghum genes in this group have also been shown to regulate male fertility in response to abiotic stress conditions. For example, *Drought-induced lipid transfer protein* (*OsDIL*), orthologous to Sobic.001G266600, affects pollen fertility under drought conditions^48^, while, rice ortholog of a glycosyl hydrolase gene (Sobic.001G099700) confers cold tolerance to developing pollen^49^. Overall, 58 genes of this group exhibit anther-preferential expression, among which, rice orthologs of four genes (*OsINV4* (*Cell wall invertase 4*), *OsDIL*, *OsNP1*, and *TDR*) have already been genetically characterized for regulating anther/pollen development. Among transcription factor families, NAC and MYB family transcription factors were particularly enriched in this group (Fig. 3).

#### Group 4 exhibits preponderance of stress-associated genes

Group 4 contains 168 genes with high expression in both A1 and A3 stages. This characteristic pattern can also be interpreted as dip in expression during microspore stage. Pathway analysis revealed many genes mapping to abiotic stress, calcium signalling pathways, glutathione S transferases, phosphatases, and invertases, and the genes encoding cytochrome P450 enriched in this group (Fig. 3, Table S4). Furthermore, 65 genes of this group exhibited anther-preferential expression. Although, rice orthologs of none of these have been characterized for roles in anther development, some of these genes have been implicated in stress response. Such as, rice ortholog (*OsABA8Ox3; ABA 8*′*-hydroxylase 3*) of a cytochrome P450 encoding gene (Sobic.007G156300) is upregulated in response to cold and ABA in anthers^50^. Also, *OsABA8Ox3* exhibits downregulation in cold sensitive genotype, which is associated with cold-induced pollen sterility^50^. Another gene, Sobic.008G058500, putatively codes for a CYP71E1 protein. CYP71E1 has been previously reported as a stress-related protein in sorghum^51^.

Glutathione S transferases and calcium signalling genes, prominent in this group, are also important components of abiotic stress-related pathways. Among the calcium signalling genes detected in this group, rice ortholog of Sobic.003G082600, *OsCML16* (*Calmodulin-like protein 16*) has been shown to enhance drought tolerance^52^. Whereas, rice ortholog of Sobic.003G365600, *MID1* (*MYB Important for Drought response 1*) plays crucial role in anther development in response to drought stress^53^.

Pollen development is also highly sensitive to heat stress^51^. Spikelet sterility due to faulty pollen development and premature anther dehiscence in response to heat stress has been well-demonstrated in rice^54,55^. Frova and co-workers^56^ observed increased expression of heat shock proteins in early as well as the late stages of pollen development in sorghum. We also observed several genes involved in heat stress tolerance in this group. These include rice ortholog (*OsHSP18*.*2*) of one of the heat shock proteins (HSP), Sobic.003G039400, which imparts heat stress tolerance to germinating seeds by restricting ROS accumulation^57^. Whereas, Sobic.003G039400 ortholog, *OsHSP17*.*0* has been shown to confer salt and drought tolerance in rice^58^. Additionally, three HSPs in this group are orthologous to *Arabidopsis HSP17*.*6* (AT1G53540), regulated by chemical heat response inducers^59^. In-depth characterization of these genes will provide candidates to combat with abiotic stress-induced male sterility in sorghum^60^.

#### Group 5 genes regulate protein degradation and cell wall modification in the late pollen stages

Group 5 contains 499 genes exhibiting gradual increase in expression from meiotic to post-meiotic stage that peaks in mature pollen stage (Fig. 3). The key pathway categories in this group are associated with protein metabolism and transport including protein degradation, peptides/oligopeptides transport, and major intrinsic proteins. Other prominent categories are lipid metabolism and cell wall modification, with the latter showing significant enrichment (Table S4, Fig. 3). Recently, Moon and co-workers^61^ also highlighted abundance of the cell wall modification genes in mature pollen of rice.

The protein degradation-related genes include subtilases, ubiquitin-related genes, cysteine proteases, serine proteases, and ATPases. While, four genes in the major intrinsic proteins category in this group code for aquaporins. Conversely, lipid metabolism-related genes code for acetyl-CoA carboxylation, beta ketoacyl-CoA synthases, acyl-CoA binding proteins, and acyl-CoA ligases. Rice ortholog of two of the genes in this group, *MID1* (*Midline 1*) and *OsINV1* (*Invertase 1*) regulate pollen development in response to abiotic stress conditions^49,53^. Several cell wall modification genes including expansin precursors, a glycosyl hydrolase family 16 gene and four genes that encode for putative pollen allergen proteins are also enriched in this group. Two of the expansin genes, Sobic.001G306200 and Sobic.001G306400, are orthologs of *OsIPA*, which encode for expansins/pollen allergens with conserved role in both rice and *Arabidopsis*^62^.

Furthermore, 99 genes of group 5 exhibit anther-preferential expression. Among these, three are orthologous to rice *OSIPA* (*Oryza sativa indica pollen allergen*) and *OsPCBP* (*Pollen specific calmodulin-binding protein*) genes involved in pollen development^62,63^. Several of the group 5 genes encode for cell wall modification-related genes encoding pollen allergens and expansins (Table S3A). Pollen expressed expansins are also known to help in cell wall deposition, pollen germination, pollen tube growth and penetration while some of the expansins belong to the category of pollen allergens^64^. Group 5 genes, therefore, likely play role in cell wall modifications during pollen development.

#### Group 6 genes regulate cell wall, phytohormone and carbohydrate metabolism in mature pollen

Group 6, exhibiting mature pollen stage preferential/specific expression, contain the maximum number of genes (2291) belonging to 17 pathway categories (Table S4, Fig. 3). Maximum number of genes in this group are implicated in protein degradation and post translational modifications. These include subtilases, ubiquitin-related genes, cysteine proteases, aspartate proteases, serine proteases, ATPases, etc. Other notable categories/pathways enriched in this group include cell wall synthesis, ethylene metabolism, abscisic acid metabolism, metal transporters, and carbohydrate synthesis. In addition, transcription factors belonging to RAV, CO-like, DBB, DOF, ERF, and TALE families are also enriched in group 6.

One of the key events during pollen maturation is pollen wall development. Pollen wall comprises an outer exine made of sporopollenin, and an inner intine which is composed of cellulose, pectin, and hemicellulose. Cellulose synthase (CESA) and synthase like (CSL) genes in *Arabidopsis* have been shown to play important roles in pollen wall development and tube growth^61,65,66^. *CESA7* ortholog, Sobic.001G224300 and eight cellulose synthase-like genes belonging to CSLD, CSLE, CSLF, and CSLH subfamilies exhibit group 6 pattern. Their increased expression from microspore to mature pollen stage corroborates with potential role in pollen wall synthesis.

Another metabolic checkpoint for pollen maturity is starch accumulation which serves as an energy resource during pollen germination^67^. Manipulation of genes involved in sugar-starch conversion can lead to starch deficiency, leading to sterility, as exemplified by the expression analysis of these genes in male sterile lines of sorghum and maize^67^. We identified nine genes involved in sucrose and starch metabolism, in addition to fifteen sugar transporters, seven genes related to trehalose metabolism, and seven genes involved in sugar and nutrient signalling, in this group. These are important candidates for regulating carbohydrate metabolism during pollen maturation. Rice ortholog of Sobic.004G013500 encodes UTP-glucose-1-phosphate uridylyltransferase (*OsUGP2*) with binucleate and mature pollen stage-preferential expression. *OsUGP2* regulates starch accumulation in pollen^68^. Conversely, rice ortholog (*WDA1; Wax-deficient anther 1*) of Sobic.001G222700 is involved in fatty acid biosynthesis in tapetum and anther walls^69^.

In addition, 17 ethylene and 11 abscisic acid metabolism-related genes including aminocyclopropane carboxylate oxidases (ACCs) and aldehyde oxidases are enriched in this group. Ethylene is important for conferring thermotolerance in pollen^70^. Also, recent findings in rice indicate that enhancement of sucrose metabolism in response to ABA signalling is crucial for pollen protection from heat stress^71^. Thus, upregulation of ethylene and abscisic acid signalling may be important for the acquisition of abiotic stress tolerance in pollen. Increase in expression of ethylene and ABA genes during pollen development has also been observed in rice^72^. Furthermore, several key genes involved in jasmonic acid, cytokinin and auxin biosynthesis, and signaling also exhibit group 6 pattern, pointing to coordinated action of different phytohormones in pollen maturation^73^ (Table S3b).

Metal transporters can be important for maintaining metal ion homeostasis in developing pollen. Li and co-workers^74^ showed that magnesium homeostasis is important for pollen development. Disruption of magnesium transporter, *AtMGT4* (*Arabidopsis Magnesium transporter 4*) leads to abnormal pollen development. We identified 17 metal transporter genes in group 6 including zinc, cadmium, copper, magnesium, manganese, and iron transporters. Furthermore, 351 genes of group 6 exhibit anther-preferential expression. These include sorghum orthologs of rice *OsMADS3* (Sobic.003G027000), *OsINV4* (Sobic.006G070032), *OsPLIM2b* (Sobic.006G159000), and *MEL2* (Sobic.008G138200) (Table 1). The male sterility of a recessive male-sterile line RI127S in rice has been attributed to loss-of-function mutation in *OsMADS3*^75^. Both *OsPLIM2b* and *OsINV4* have been shown to exhibit mature pollen-specific expression and regulate anther development^49,76^. *MEL2* (*Meiosis Arrested at Leptotene 2*), on the other hand, is involved in translational regulation of several meiotic genes in rice^77^.

#### Group 7 genes regulate secondary metabolite biosynthesis in microspore and mature pollen stages

Group 7 contains 151 genes exhibiting increase in expression from meiotic to post-meiotic stage that sustains in mature pollen stage as well (Fig. 3). Genes belonging to NAC family of transcription factors and those encoding for secondary metabolites are particularly enriched in this group (Fig. 3). Eighty-one genes of group 7 are anther-preferential, however, rice orthologs of none of them have been functionally characterized, till date.

### Comparative genomic analysis in rice and sorghum emphasize conserved developmental pathways regulating anther development

Though the molecular regulation of anther and pollen development has been well elucidated in several model plants^78–81^, little is known about these processes in sorghum. Comparative genomic approaches provide an opportunity to translate this knowledge in agriculturally important crop plants^31^. However, mere sequence homology is not enough to predict functional conservation among orthologous genes. Comparative expression profiling in analogous stages of development is necessary to identify the functional orthologs with conserved roles^31^.

**Figure 4:**
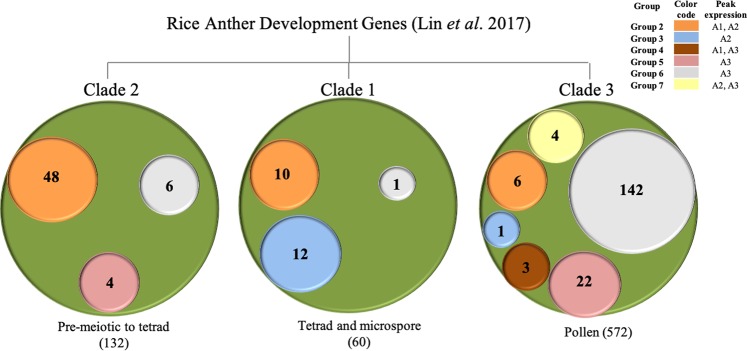
Comparison of sorghum and rice anther transcriptome. Lin and co-workers^82^ identified differentially expressed genes (DEGs) in several male sterile mutants of rice and divided them into three clades based on stage specific expression. Clade 2, 1, and 3 represent premeiotic to tetrad, tetrad and microspore, and pollen-related genes, respectively. A comparison with different groups of DEGs in our data highlighted interesting parallels between two studies. Based on orthology, the clade 2 genes of rice showed maximum common genes with group 2 of our data; clade 1 showed common genes with groups 2 and 3; and clade 3 showed maximum common genes with groups 5 and 6.

Recently, Lin and co-workers^82^ generated a co-expression network from different developmental stages of anthers, named RiceAntherNet, by compiling data from 57 microarrays. Using hierarchical clustering of DEGs from nine male sterile mutants corresponding to master regulators of anther development viz., *MSP1* (*Multiple sporocyte 1*), *TDL1*, *GAMYB* (*Gibberellin MYB*), *TIP2*, *UDT1* (*Undeveloped tapetum 1*), *TDR*, *EAT1*, *PTC1* (*Persistent tapetal cell 1*), and *MADS3*, authors identified 29 gene clusters which could be classified into three clades: clade 1 genes specifically expressed in stage 8 and 9 representing tetrads and uninucleate microspore stage, clade 2 genes expressed in stages 2 to 8 harbouring pre-meiotic to tetrad stage anthers, whereas, clade 3 genes expressed in mature pollen (Fig. 4). Out of 764 rice genes comprising clade 1, 2, and 3 of RiceAntherNET, sorghum orthologs of 258 genes were differentially expressed in sorghum anthers as well. Comparison of the gene clusters obtained in our study for sorghum vis-à-vis clades identified in RiceAntherNet revealed interesting parallels (Fig. 4; Table S6). The clade 2 of RiceAntherNet possessed 132 genes. Of these, sorghum orthologs of 48 genes exhibited group 2 pattern. Clade 2 of RiceAntherNet as well as group 2 genes of sorghum in our study exhibited peak expression in premeiotic to tetrad stages of anther development. Similarly, out of 60 clade 1 genes of RiceAntherNet, 22 genes belonged to group 2 and 3 of sorghum, with both these groups exhibiting high expression in tetrad to microspore stages. Furthermore, out of 572 clade 3 genes of RiceAntherNet, sorghum orthologs of 142 genes belonged to group 6, with both clade 3 and group 6 having pollen-specific expression. High concurrence in expression patterns of genes during anther development in rice and sorghum confirm conserved developmental pathways underlying male gametophyte development in grasses.

In fact, Cigan and co-workers^7^, recently demonstrated conserved role of *SbMs26* (Sobic.001G491400), orthologous to rice *CYP704B2*, in regulating male fertility. Targeted mutagenesis of this gene led to defects in pollen formation. Therefore, collating information about rice orthologs of differentially expressed genes in sorghum anthers is a valid strategy for shortlisting candidates for engineering male fertility in sorghum. Rice orthologs of 63 differentially expressed genes in sorghum anthers have been functionally characterized for their involvement in anther/pollen development using forward and/or reverse genetics approaches (Table 1). Among these, 21 genes exhibit anther-preferential expression and therefore, would serve as important targets for engineering male fertility without off-target effects.

### Analysis of *cis*-regulatory elements

The spatial and temporal specificity of gene expression is mediated by specific binding of transcription factors on *cis*-regulatory elements. Therefore, analysis of *cis*-regulatory elements is essential to elucidate mechanistic basis of gene regulation. Several *cis*-regulatory elements have been earlier associated with genes exhibiting anther and/or pollen-specific expression in model species, rice and *Arabidopsis*^83–87^. Based on the published literature, we compiled a list of 65 such elements and queried them separately in 1 kb regulatory regions of differentaily expressed genes. The enrichment analysis was performed separately for each group using regulatory regions of all the genes annotated in sorghum as reference. Notably, 41 out of 65 elements analysed exhibited significant enrichment in the regulatory regions of differentially expressed genes in sorghum anthers (Supplementary Table S7). Most of them have been identified from regulatory regions of tapetum and/or male gamete-specific genes in rice (Supplementary Table S7). Among unique motifs enriched in regulatory regions of group 1 genes is MSACRCYM motif which has earlier been reported from promoter of cell cycle-related genes in *Catharanthus*^84^. This is particularly interesting because cell cycle-related genes are also enriched in group 1 (Fig. 3). An ABA-responsive motif ABRE3HVA22 is specifically enriched in regulatory regions of group 6 genes which are also enriched in ABA biosynthesis/signalling-related genes^84^ (Supplementary Table S7 and Fig. 3). Interestingly, group 7 genes exhibit enrichment of 5659BOXLELAT5659 element which has been shown to be responsible for pollen specific expression of pollen expressed genes, LAT56 and 59, of tomato^84^. These findings hint to a conserved regulatory mechanism for cell/tissue/developmental stage-specific expression of genes regulating male gametophyte development in plants.

### Validation of RNA-seq data by qRT-PCR analysis

**Figure 5:**
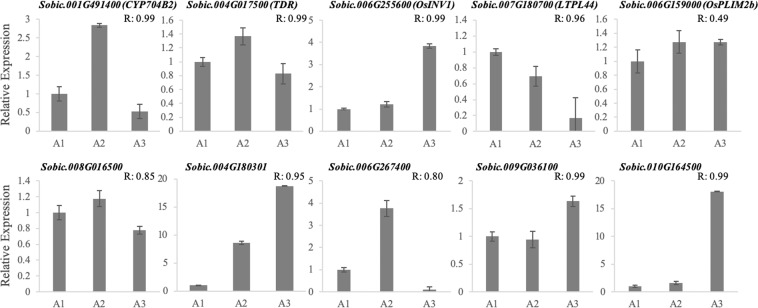
Relative expression of ten genes in anther stages measured by qRT-PCR. Expression of ten genes exhibiting varied expression patterns in RNA sequencing data was analyzed using qRT-PCR. The locus IDs of genes are given on the top with corresponding rice orthologs (if characterized). The Pearson’s correlation coefficient (R) value for each gene indicating correlation between qRT-PCR and RNA-seq data is given on the top right corner.

To validate the expression patterns of differentially expressed genes in the three anther stages, we selected ten representative genes exhibiting varied expression patterns (Supplementary Table S8). Rice orthologs of five of these genes have been earlier shown to perform crucial functions during anther development in rice (Table 1). Except one gene which exhibited Pearson’s correlation coefficient of 0.49 with the RNA-seq data, expression patterns of all other genes showed good correlation ranging from 0.80 to 0.99 between two techniques, indicating high reproducibility of the transcriptomic data (Fig. 5).

**Figure 6:**
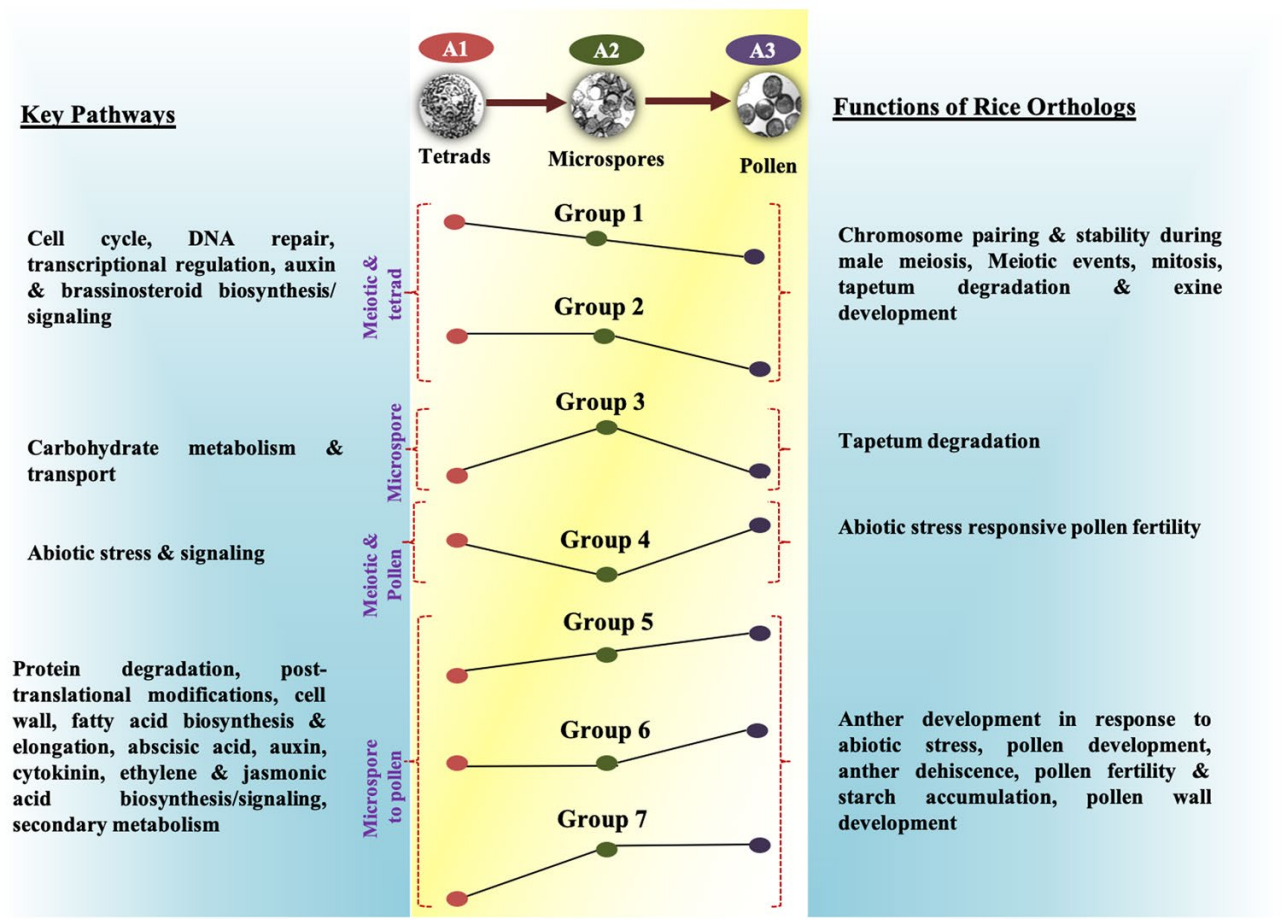
Overview of dominant patterns and associated functions. Four different types of patterns representing premeiotic to tetrad stage preferential expression, microspore-preferential, early and late pollen-preferential and mature pollen preferential patterns are shown in the middle. The key pathways associated with each category are shown on the left, while right panel indicating key functions exhibited by rice orthologs of sorghum genes identified in these categories.

Overall, transcriptional trajectories collated with enriched pathways and functions of rice orthologs depict conserved molecular cascade underlying progression of anther development in sorghum (Fig. 6). The genes belonging to these cascades will serve as valuable resource for engineering male sterility in sorghum with potential applications in hybrid breeding, eliminating pollen allergens and avoiding gene flow from genetically modified plants. The expression of ribonuclease *barnase* genes driven by anther/pollen-specific promoter has been extensively used for engineering male sterility in other crop plants^88–90^. Therefore, the regulatory elements of shortlisted 21 anther-preferential genes would be important candidates for driving tissue-specific expression. To facilitate this, anther expressed genes can be experimentally validated for their tissue specificity using reporter genes. Another strategy would be to engineer male sterility by targeted mutagenesis of anther-specific genes using CRISPR/cas9 or gene silencing strategies. The genetically engineered male sterile lines would then be assessed for genetic stability, heterosis and, potential linkage with unwanted traits using empirical approaches^91,92^. Recently, we have reported small RNA profiles of meiotic and post-meiotic anthers in sorghum. Interestingly, several of the differentially expressed genes in anthers are also miRNA targets providing additional opportunity to engineer male sterility by targeting non-coding RNAs^93,94^.

## Methods

### Staging and harvesting of anther samples

*Sorghum bicolor* variety M35-1 plants were grown at the ICAR-Indian Agricultural Research Institute, New Delhi, India. The panicles were collected at different stages of development based on the position of flag leaf ranging from pre-emergence to fully emerged panicles, so as to harvest the anthers involving meiotic to mature pollen stages. The length of anthers, dissected from panicles at different stages of development, were measured and recorded. Freshly extracted anthers, based on their length, were categorized into three stages and stored in separate tubes containing TRIzol^TM^ reagent with stage A1 containing anthers of lengths smaller than 1.5 mm, stage A2 containing 1.6 to 2.0 mm anthers and, stage A3 containing 2.5 to 3.0 mm anthers. Three replicates of anthers collected for each stage were snap frozen in liquid nitrogen and stored at −80 °C. About 15–20 anthers from each stage were simultaneously stored in a fixative (ethanol and glacial acetic acid in 3:1 ratio) at 4 °C for microscopic analysis.

### Microscopic analysis

To confirm the staging of anther development, microscopic analysis was performed with the anthers sampled from each stage (A1-A3) using a published protocol with minor modifications^95^. Briefly, anthers were dehydrated using ethanol gradient (50%, 80%, 90% and 100%) for 1 hour each followed by two washes of 100% and 90% acetone for one hour each. Anthers were cleared in 100% and 90% xylene for one hour each and, passed through two changes of paraffin wax at 75 °C for 1.5 hours each. Further, 5 µm sections were obtained from the embedded tissue using a Leica RM2235 microtome (Leica, Germany). Samples were deparaffinized in xylene and again passed through graded ethanol series for 2–3 minutes each. Finally, the sections were washed in running tap water and stained with toluidine blue O for 15–20 seconds. Excess stain was removed by rinsing with tap water. Sections were dried, mounted using resinous mounting medium and viewed using bright field microscope (Nikon, Eclipse 80i).

### RNA isolation, library preparation and sequencing

Total RNA was extracted from anther tissue using TRIzol^TM^ reagent as per manufacturer’s instructions (Invitrogen) with minor modifications. Briefly, anthers suspended in 200 µl of TRIzol^TM^ were crushed finely using epi-pestles. To the grinded sample, 800 µl of TRIzol^TM^ was added and mixed by inverting the tubes. After 5 min incubation at room temperature (RT), tubes were centrifuged at 10,000 rpm for 10 min at RT to remove the debris. To the supernatant, 200 µl of chloroform was added followed by centrifugation at 13000 rpm for 10 min at RT. To the supernatant, half volume of 7.5 M ammonium acetate was added and mixed gently. Samples were centrifuged at 12000 rpm for 10 min. To the supernatant collected in fresh tube, 3 volumes of chilled ethanol was added, and tubes were stored at −20 °C overnight for RNA precipitation. Next day, samples were centrifuged at 10,000 rpm for 5 min at 4 °C. The pellet was washed twice with 75% chilled ethanol, dried at RT and dissolved in RNAse free water. The quantity and quality of RNA samples were determined using agarose gel electrophoresis, Nanodrop^TM^ 2000 and Agilent 2100 Bioanalyzer. Only RNA samples with RIN value >8 were used for library preparation. The sequencing libraries were prepared using TruSeq^®^ Stranded Total RNA kit using manufacturer’s instructions and sequenced using Illumina HiSeq 2500 paired-end sequencing with an average read length of 100 bp. The RNA-seq data has been submitted to NCBI Gene Expression Omnibus (GEO) and can be accessed using its assigned accession number, GSE141035.

### Data analysis

Low-quality reads with Phred score <30 were filtered and adapters were trimmed using AdapterRemoval version 2.2.0^96^. From the pre-processed reads, ribosomal RNA sequences were removed by aligning the reads with SILVA database^97^ using Bowtie version 2.2.9 with default parameters. Reference genome sequence and annotations of sorghum were downloaded from Phytozome (URL: https://genome.jgi.doe.gov/portal/pages/dynamicOrganismDownload.jsf?organism=Sbicolor). High quality reads were aligned to the sorghum genome using STAR aligner^98^ using default parameters. The aligned reads were used for estimating expression of the genes and transcripts using StringTie version 1.3.3b^99^. The correlation between biological replicates was calculated using Spearman’s correlation coefficient using R.

Pairwise differential expression analyses between different developmental stages was performed using cuffdiff program of cufflinks package^100^. The transcripts with log_2_ fold change ≥1 (up-regulated genes) or ≤−1 (down-regulated genes) and q-value cut off ≤0.05 were considered as differentially expressed. FPKM values of differentially expressed transcripts were used for clustering the data using K-means clustering in MultiExperiment Viewer (MeV)^101^. Pathway analysis was carried out using MapMan^102^ with Sbicolor_79 1.0 mapping file (https://mapman.gabipd.org/mapmanstore), and enrichment of pathway sub-categories was determined using hypergeometric test (p-value ≤0.05). Further, p-values were corrected using Benjamini-Hochberg multiple-testing correction (false discovery rate (FDR) < 0.05). The information about transcription factors in sorghum was extracted from ‘Plant Transcription Factor Database’^103^ and used for enrichment analysis using hypergeometric test (p value ≤0.05). The p-values were corrected using Benjamini-Hochberg test (FDR < 0.05). The information of the rice as well as *Arabidopsis* orthologs was obtained from Phytozome v3.1.1 (https://phytozome.jgi.doe.gov/pz/portal.html). Rice genes, previously characterized for their role in anther development, were extracted from FunRiceGenes database^104^ (URL: https://funricegenes.github.io/). The phytohormone-related genes from rice were obtained from published literature^28^ and the information about their sorghum orthologs was extracted from Phytozome v3.1.1 (https://phytozome.jgi.doe.gov/pz/portal.html). To identify anther-preferential genes, publicly available expression data from 47 RNA sequencing experiments corresponding to different stages of root, shoot, leaf, panicle and seed development as well as stress treatments, was downloaded using “PhytoMine” tool available at Phytozome database. The genes exhibiting FPKM > 1 in any of the vegetative or seed stages with or without treatment were filtered from differential expressed gene set to extract anther-preferential genes.

### Analysis of *cis*-regulatory regions

To assess the enrichment of known motif elements in the regulatory regions of differentially expressed genes in anthers, sequences corresponding to 1 kb regulatory regions of all the differentially expressed genes were downloaded using BioMart tool of Phytozome (https://phytozome.jgi.doe.gov/biomart/martview/f7080098cbb5de1a01647b16a4422139). The list of 65 known motifs implicated in anther/pollen development was compiled from literature^83–87^ and separately queried in the regulatory regions of genes belonging to each cluster of DEGs using CentriMo tool under MEME suite (http://meme-suite.org/tools/centrimo) with default parameters. For enrichment analysis, dataset of 1 kb promoter regions of all sorghum genes was used as reference.

### qRT-PCR analysis

RNA was extracted from three biological replicates of each anther stage as described above. cDNA was prepared using iScript™ cDNA Synthesis Kit (Biorad) and qPCR was carried out using EvaGreen dye (G-Biosciences) on a CFX96 Real Time System (BioRad) following manufacturer’s instructions. Briefly, a 10 μL reaction was prepared using 5 μL EvaGreen dye, 1 μL primer (each primer), 1 μL of diluted cDNA template and 2 μL of nuclease free water. Reaction conditions were as follows: 95 °C for 2 mins, followed by 40 cycles of 15 s at 95^◦^C and 30 s at 60 °C. After amplification, melt curves were analysed to ensure primer specificity in each reaction. Relative transcript expression levels were obtained using the double delta C_T_ (ΔΔC_T_) method. Eukaryotic Initiation Factor 4α (eIF4α) was used as an internal control^105,106^. The expression values were expressed as mean ± SD. The primers used for the qRT-PCR are listed in Supplementary Table S7. Pearson’s correlation coefficient (R) was calculated between the expression values obtained using RNA sequencing and qRT-PCR analysis for each gene.

## Supplementary information

Supplementary information
